# Unusual Presentation of Coxsackievirus B and Methicillin-Sensitive Staphylococcus aureus Cellulitis Causing Sepsis

**DOI:** 10.7759/cureus.47826

**Published:** 2023-10-27

**Authors:** Britton A Ethridge, Cory J Dixon, Paul Q Vu, Michael B Steadman, Aaron P Tillman, Natalie S Barefield, Matthew C Ragan

**Affiliations:** 1 Department of Research, Alabama College of Osteopathic Medicine, Dothan, USA; 2 Department of Primary Clinical Skills, Alabama College of Osteopathic Medicine, Dothan, USA; 3 Department of Obstetrics and Gynecology, Aventa Specialized Women's Care, Dothan, USA

**Keywords:** international travel, hypotension, pancytopenia, edema, lymphangitis, cellulitis, mssa, sepsis, vector borne disease, coxsackie b virus

## Abstract

The clinical association between Coxsackievirus B (CVB) and methicillin-sensitive *Staphylococcus aureus* (MSSA) has not been well established in the current literature. Here, we report a case of a 29-year-old male who presented with fever and malaise 24 hours after noticing a pruritic lesion on the anterior foreleg that resembled a mosquito bite. After multiple ED visits, laboratory studies, and imaging tests, the patient was admitted for treatment of high fevers and pancytopenia. The final diagnosis was viral sepsis complicated by co-infection with MSSA.

## Introduction

Coxsackievirus B (CVB) is a positive-sense, non-enveloped, single-strand RNA enterovirus of the Picornaviridae family that includes six different serotypes (B1-6) which are responsible for numerous clinical presentations [1-3]. Most enteroviruses, including CVB, are transmitted via the fecal-oral route [4,5]. Common symptoms include sore throat, fever, gastrointestinal distress, angina, and extreme lethargy [6]. The incubation period is two to six days, typically progressing as a self-limiting disease within 10 days for healthy adults. However, some cases have been reported to last more than six months [7]. Symptom severity depends on the immune status and age of the individual affected, with nearly all severe or lethal cases occurring in neonates or the immunocompromised [2]. CVB is not associated with any specific racial, gender, or ethnic group [6]. CVB infections frequently occur during the summer months [2,5,6]; however, in tropical regions, CVB seems to propagate year-round [8].

Methicillin-sensitive *Staphylococcus aureus* (MSSA) is a clinically pertinent gram-positive cocci [9], which is frequently seen in both the clinical and community environment [10]. Because MSSA is a normal flora of the nares, skin, and gastrointestinal infection, it does not usually cause symptomatic infection [11-13]. When it does, MSSA infection can present with various manifestations ranging from minor skin infections to endocarditis and sepsis [10,14]. One meta-analysis cited the mortality rate of MSSA bacteremia as 23.2% [15].

Co-infection of CVB and *Staphylococcus aureus* (MSSA and MRSA) is rarely reported in the literature, and in the cases available, presentation varied widely [16,17]. Here, we report a case of co-infection of CVB and MSSA cellulitis causing sepsis.

This article was previously presented in the poster competition at the 2023 American Medical Association - Medical Student Section Physicians of the Future Summit on January 28, 2023.

## Case presentation

**Figure 1:**
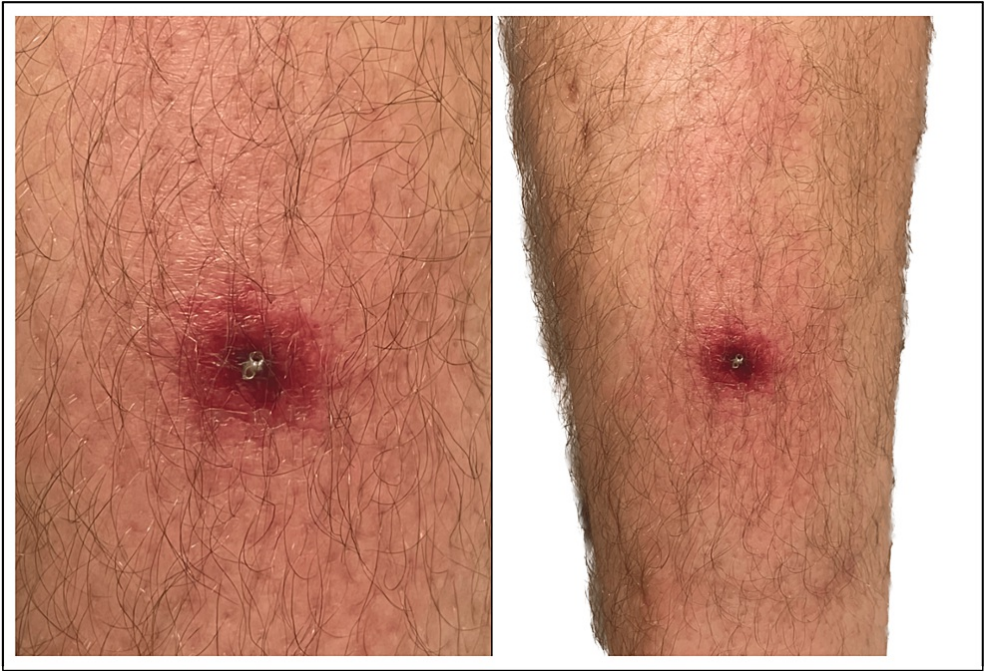
1-2 cm pruritic, erythematous lesion exhibiting a necrotic core on the left anterior foreleg

A 29-year-old male was traveling in the state of Virginia when he first noticed a 1-2 cm reddened pruritic lesion on the anterior foreleg, resembling a mosquito/spider bite (Figure 1). By the evening of day 1, the patient developed a temperature of 99.8F and malaise, which prompted a telehealth visit to his primary care provider. The patient was reassured that his condition was most likely self-limiting but advised to go to the ED if symptoms worsened.

**Figure 2:**
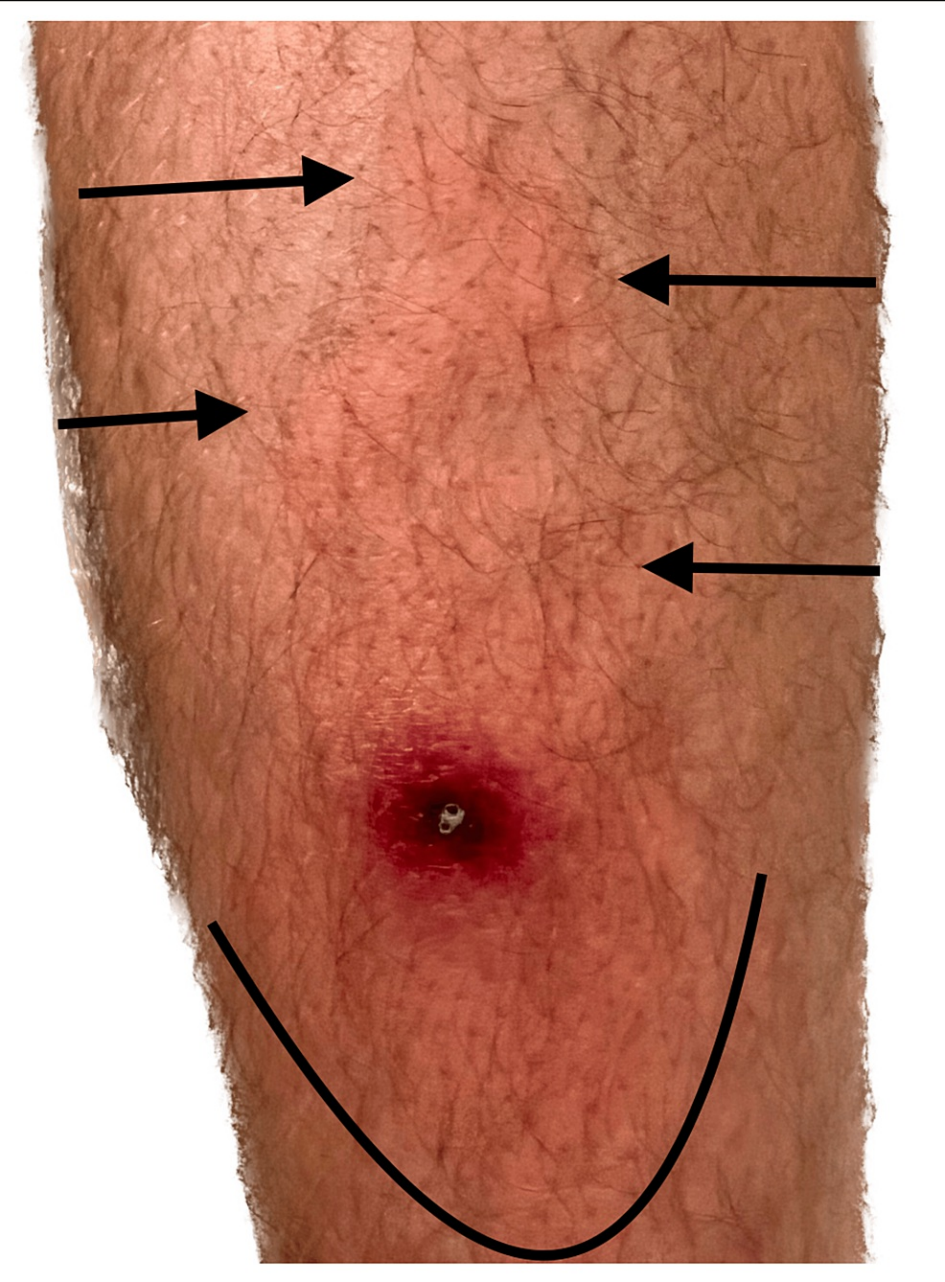
Lesion exhibiting lymphangitis/cellulitis and beginning to advance cephalad toward the groin

Within two days of symptom onset, the lesion border has advanced to a 5-6 cm diameter and developed progressive ascending warmth, redness, and generalized limb discomfort (Figure 2). Continued changes in lesion appearance, malaise, and fever warranted an ED evaluation by the end of day 2. The patient had worsening lymphangitis/cellulitis but was afebrile and normotensive. A scant amount of lesion exudate was expressed and cultured. Because of suspicion of staphylococcal skin infection, trimethoprim/sulfamethoxazole (800/160) BID was prescribed for 10 days while awaiting culture results.

**Figure 3:**
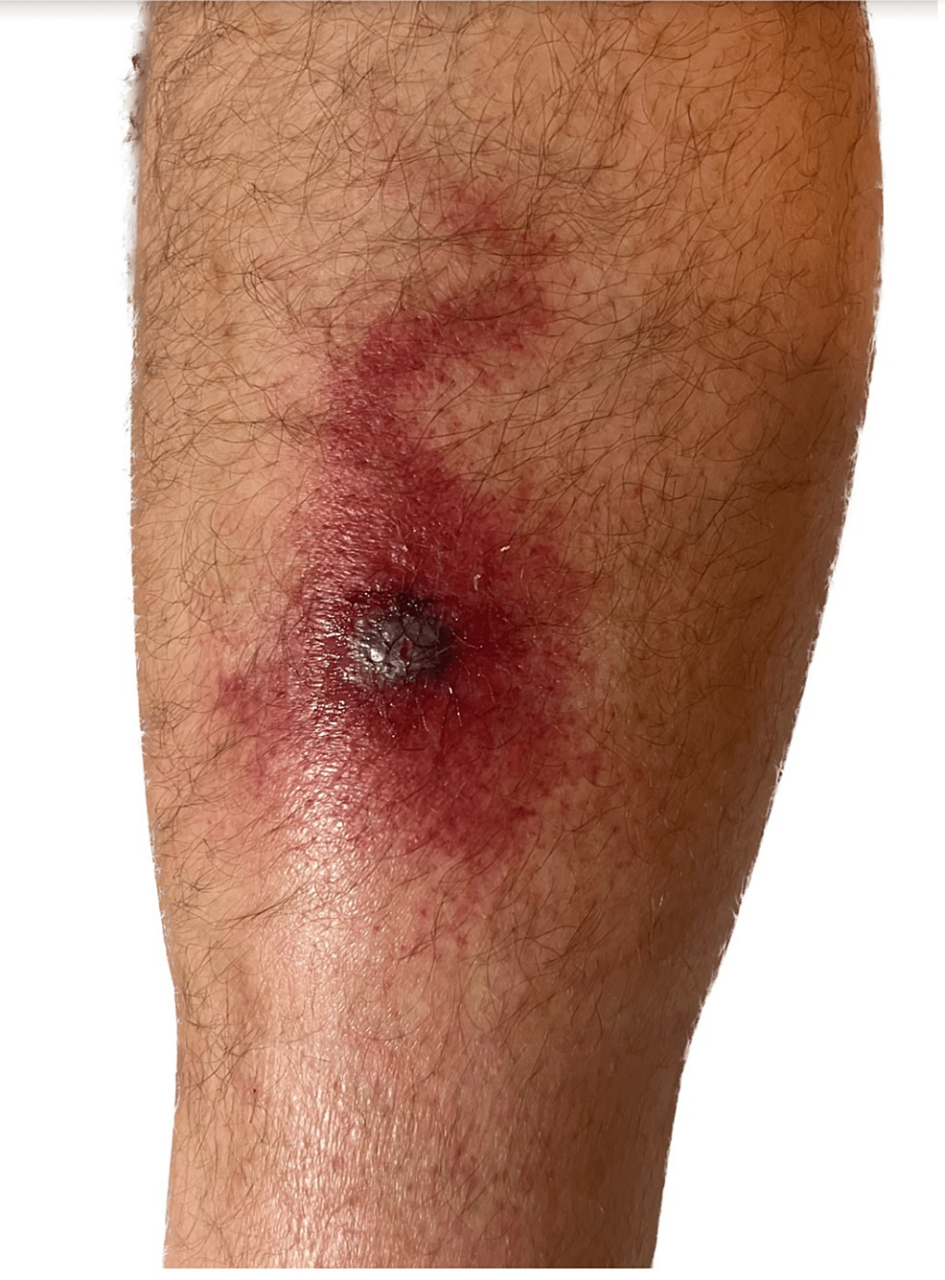
Worsening lymphangitis, cellulitis, and new onset of 2+ pitting edema of the lesion seen on day 7. Notice the central weeping and change in swelling at the bottom of the image after the patient removed his sock

Five days later, the patient traveled eight hours by car and developed 2+ pitting edema, mild serosanguineous wound drainage, and increased pain but remained afebrile (Figure 3).

**Figure 4:**
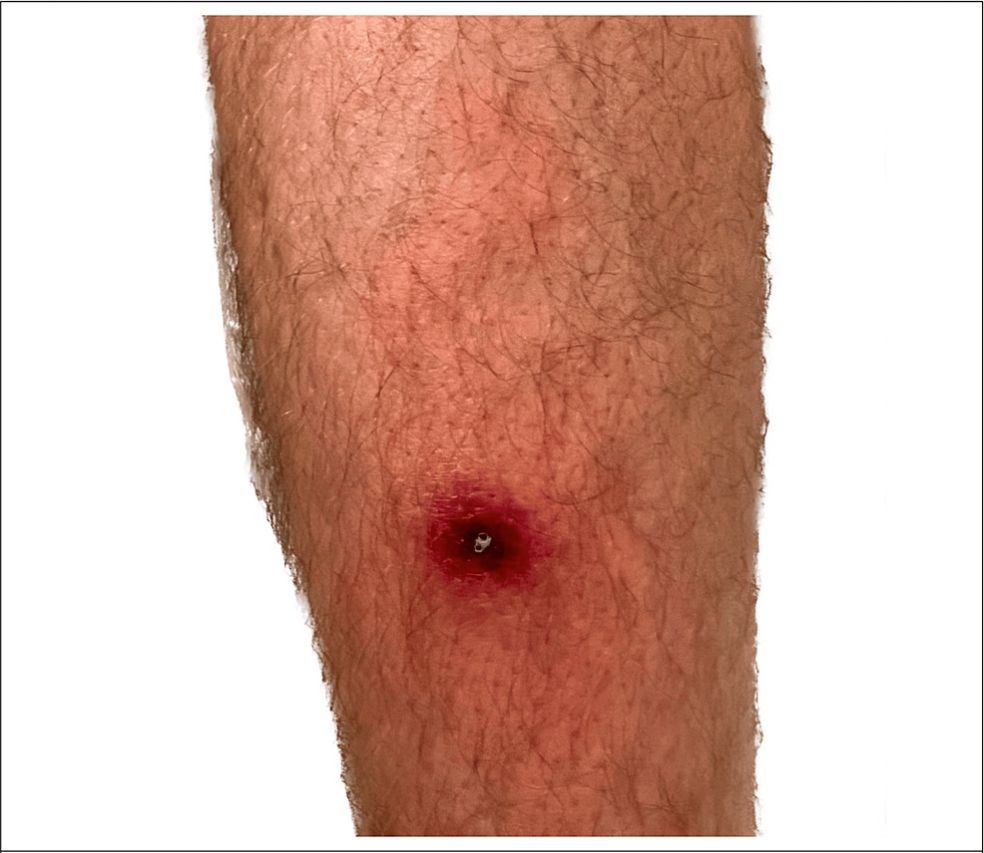
Lesion appearance showing improving cellulitis and edema on day 9

The patient applied compression stockings and elevated his legs. Within six hours, the patient developed a fever of 103.9 F, chills, and severe malaise. The patient self-administered acetaminophen 1000 mg every six hours (q6h), which decreased his temperature by about 1 degree when in full effect. Despite visible lesion improvement, the patient continued to experience moderate retro-orbital headache, difficulty with focusing/cognition, and severe lethargy (Figure 4). Two days later, the patient returned to the ED with the following vital signs: temperature 102.2F, heart rate 100, blood pressure 131/69, oxygen saturation 97% on room air, and respiratory rate 15 but was soon discharged after being switched to doxycycline 100 mg BID due to suspicion of inadequate antibiotic coverage (Table 1).

**Table 1:** Vital signs taken at the ED visit on day 9

	July 17, 0933
Temperature (oral)	38.9 C
Heart rate	100
Respiratory rate	15
Blood pressure (systolic)	131
Blood pressure (diastolic)	69
Oxygen saturation	97%

**Figure 5:**
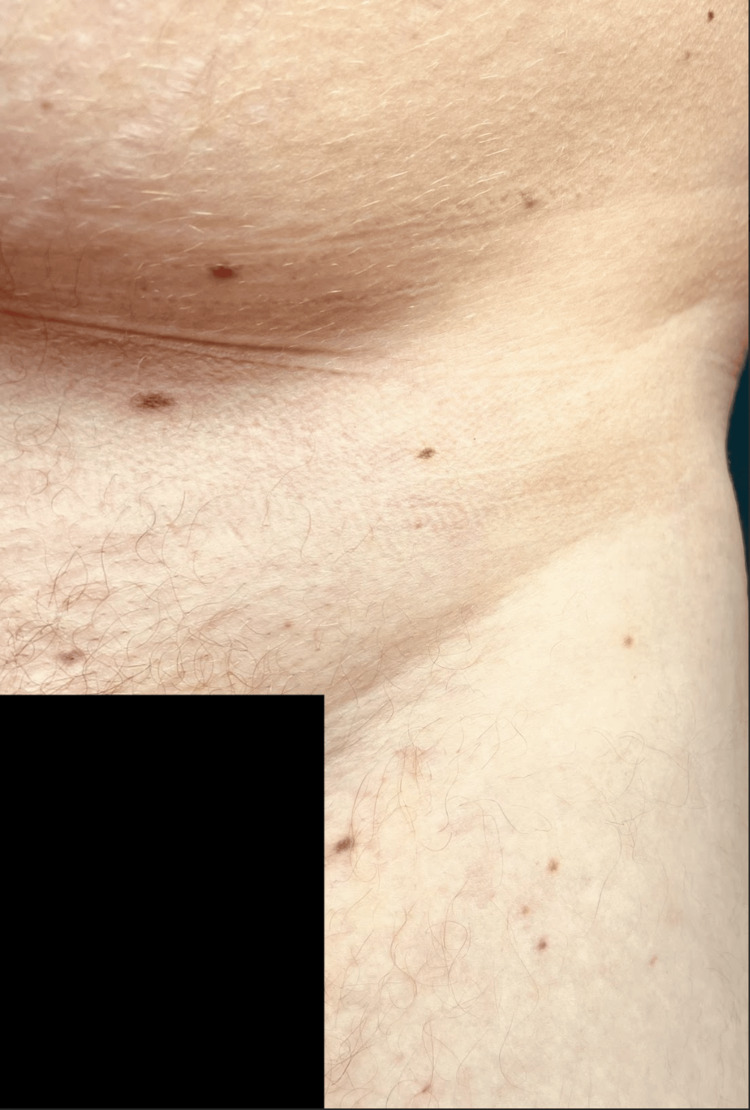
Flat, non-palpable, red-purple, painless, subcutaneous lesions consistent with CVB seen on the inguinal region. Isolated lesions were also found on the left palm and buccal mucosa

Less than 24 hours later (day 10), the patient was admitted for antibiotic failure with weakness, blood pressure lability, lymphocytopenia, and witnessed syncope in the ED. Bedside arterial blood gases were notable for PaO2 of 28 mmHg, with all other standard values within normal ranges. CBC revealed a total WBC 5.86 x10-3/uL, platelets 142,000 per microliter (mcL), neutrophils 80.5%, lymphocytes 9.2%, and 7.8% eosinophils (Table 2). Vancomycin 2500 mg was started, followed by 1750 mg q12h infusion. On day 11, new, flat, red-purple, painless, subcutaneous lesions developed, and ceftaroline 600 mg q12h was added (Figure 5). Vital signs showed persistent hypotension and fever, and laboratory studies showed continued pancytopenia, hypocalcemia, and mild anemia. On day 11, the patient described a new sensation of extreme heat in both hands, perioral tingling, and anxiety (Tables 3-4). Infectious disease consultation was sought, and during the patient interview, a history of travel to the Dominican Republic four weeks prior was elicited. This prompted the collection of tick-borne/arbovirus panels due to concerns about tropical vectors, which had previously been deemed unlikely.

**Table 2:** Selected inpatient daily labs Abs: absolute, ALT: alanine transaminase, AST: aspartate transaminase, BMP: basic metabolic panel, BUN: blood urea nitrogen, CBC: complete blood count, CMP: comprehensive metabolic panel, eGFR: estimated glomerular filtration rate, MCV: mean corpuscular volume, MCH: mean corpuscular hemoglobin, MCHC: mean corpuscular hemoglobin concentration, MPV: mean platelet volume, RDW: red cell distribution width, SGOT: serum glutamic-oxaloacetic transaminase, SGPT: serum glutamate pyruvate transaminase

Jul 20, 1547				Jul 21, 1418			
BMP		CBC		CMP		CBC	
eGFR	>60.0 mL/min/1.73 m2	White blood cells	2.93x10-3/uL	eGFR	>60.0 mL/min/1.73 m2	White blood cells	2.76x10-3/uL
BUN/creatinine ratio	10 mg/dL	Red blood cells	4.15x10-6/uL	BUN/creatinine ratio	10 mg/dL	Red blood cells	4.27x10-6/uL
Osmolality calc	270 mosm/kg	Hemoglobin	12.5 g/dL	Osmolality calc	281 mosm/kg	Hemoglobin	13.2 g/dL
Sodium	135 mEq/L	Hematocrit	35.4%	Sodium	139 mEq/L	Hematocrit	36.6%
Potassium	4.1 mEq/L	MCV	85.3 fL	Potassium	3.9 mEq/L	MCV	85.7 fL
Chloride	103 mEq/L	MCH	30.1 pg	Chloride	105 mEq/L	MCH	30.9 pg
Carbon dioxide	22.0 mEq/L	MCHC	35.3 g/dL	Carbon dioxide	23.0 mEq/L	MCHC	36.3 g/dL
BUN	11 mg/dL	RDW	12.5%	BUN	10 mg/dL	RDW	12.4%
Creatinine, serum	1.1 mg/dL	Platelets	106x10-3/uL	Creatinine, serum	0.9 mg/dL	Platelets	111x10-3/uL
Glucose	106 mg/dL	MPV	10.5 fL	Glucose	169 mg/dL	MPV	10.2 fL
Calcium	8.1 mg/dL	Neutrophils	44.5%	Calcium	8.5 mg/dL	Neutrophils	43.7%
Anion gap	10 mEq/L	Lymphocytes	38.9%	Anion gap	11 mEq/L	Lymphocytes	42.9%
Iron, serum	42 mcg/dL	Monocytes	8.2%	Albumin	3.9 gm/dL	Monocytes	6.3%
Iron binding capacity	211 mcg/dL	Eosinophils	7.8%	Bilirubin, total	0.3 mg/dL	Eosinophils	7.1%
Iron saturation	20%	Basophils	0.3%	AST (SGOT)	21 U/L	Basophils	0%
		Neutrophils, Abs	1.3x10-3/μL	ALT (SGPT)	20 IU/L	Neutrophils. Abs	1.21x10-3/μL
		Lymphocytes, Abs	1.14x10-3/μL	Alkaline phos	53 IU/L	Lymphocytes, Abs	1.18x10-3/μL
		Monocytes, Abs	0.24x10-3/μL	Protein, total	6.6 g/dL	Monocytes, Abs	0.17x10-3/μL
		Eosinophils, Abs	0.23x10-3/uL			Eosinophils, Abs	0.20x10-3/uL
		Basophils, Abs	0.01x10-3/μL			Basophils, Abs	0.01x10-3/μL

**Table 3:** ED and inpatient vital signs from day 10

	Jul 19, 0118	Jul 19, 0252	Jul 19, 0933	Jul 19, 1329	Jul 19, 2354
Temperature (oral)	99.3 F	101.8 F	100.4 F	102.6 F	103.1 F
Heart rate	82	117	90	84	97
Oxygen saturation	96%	99%	98%	98%	95%
Blood pressure (systolic)	145	86	104	113	97
Blood pressure (diastolic)	81	56	62	58	53
Respiratory rate	14	16	11	19	20

**Table 4:** ED and inpatient vital signs from days 11 and 12

	Jul 20, 0821	Jul 20, 1149	Jul 20, 1933	Jul 21, 0321	Jul 21, 0728	Jul 21, 1322
Temperature (oral)	100.0 F	98.6 F	101.3 F	102.2 F	98.8 F	98.3 F
Heart rate	81	67	88	92	78	68
Oxygen saturation	94%	97%	98%	96%	98%	98%
Blood pressure (systolic)	115	134	108	103	115	130
Blood pressure (diastolic)	58	75	67	59	64	83
Respiratory rate	18	16	17	18	15	14

Therapy continued as previously stated until day 13, when the patient became normotensive and afebrile overnight. He was discharged on day 13 and told to follow up in three weeks for blood work (Tables 5-6).

**Table 5:** Coxsackie B1-6 titers Interpretive criteria: <1:8 antibodies not detected, > or =1:8 antibodies detected

Coxsackie B1-6 antibodies			
Component	Value	Standard range	Flag
Coxsackie B1 antibodies	<1:8	<1:8	
Coxsackie B2 antibodies	<1:8	<1:8	
Coxsackie B3 antibodies	1:8	<1:8	H
Coxsackie B4 antibodies	<1:8	<1:8	
Coxsackie B5 antibodies	<1:8	<1:8	
Coxsackie B6 antibodies	1:8	<1:8	H

**Table 6:** Three-week follow-up CMP and CBC A/G ratio: albumin/globulin ratio, CMP: comprehensive metabolic panel, eGFR: estimated glomerular filtration rate, BUN: blood urea nitrogen, AST: aspartate transaminase, SGOT: serum glutamic-oxaloacetic transaminase, ALT: alanine transaminase, SGPT: serum glutamate pyruvate transaminase, CBC: complete blood count, MCV: mean corpuscular volume, MCH: mean corpuscular hemoglobin, MCHC: mean corpuscular hemoglobin concentration, RDW: red cell distribution width, MPV: mean platelet volume, Abs: absolute

Aug 19, 1440			
CMP		CBC	
eGFR	>60.0 mL/min/1.73 m2	White blood cells	4.7x10-3/uL
BUN/creatinine ratio	9.80 mg/dL	Red blood cells	4.93x10-6/uL
Osmolality calc	277.1 mosm/kg	Hemoglobin	15.0 g/dL
Sodium	139 mEq/L	Hematocrit	42.5%
Potassium	3.8 mEq/L	MCV	86.1 fL
Chloride	105 mEq/L	MCH	30.4 pg
Carbon dioxide	26.0 mEq/L	MCHC	35.3 g/dL
BUN	10 mg/dL	RDW	13.6%
Creatinine, serum	1.02 mg/dL	Platelets	177x10-3/uL
Glucose	107 mg/dL	MPV	8.2 fL
Calcium	9.5 mg/dL	Neutrophils	57.8%
Anion gap	8 mEq/L	Lymphocytes	33.7%
Albumin	4.5 gm/dL	Monocytes	5.4%
Bilirubin, total	0.4 mg/dL	Eosinophils	2.5%
AST (SGOT)	21 U/L	Basophils	0.6%
ALT (SGPT)	30 IU/L	Neutrophils, Abs	2.7x10-3/μL
Alkaline phos	54 IU/L	Lymphocytes, Abs	1.6x10-3/μL
Protein, total	7.0 gm/dL	Monocytes, Abs	0.3x10-3/μL
Globulin, total	2.5 g/dL	Eosinophils, Abs	0.1x10-3/uL
A/G ratio	1.8	Basophils, Abs	0.0x10-3/μL

## Discussion

This patient was eventually diagnosed with viral sepsis secondary to CVB, complicated by co-infection with MSSA. Clinical identification and management of MSSA and CVB co-infection can prove challenging because both of these pathogens can present with a wide range of symptoms and severity [1-3,10,14]. In this patient, titers were significant for Coxsackie B3 and B6, which both have been shown to cause myopericarditis, aseptic meningitis, herpangina, and many other sequelae [18-20]. Additionally, MSSA can lead to various manifestations, ranging from minor skin infections to endocarditis and sepsis [10,14]. The patient’s clinical status appeared stable and mild for the initial five days before applying compression stockings, which immediately preceded a change in vital signs. The rapid change in temperature and new symptom onset suggest that rapidly mobilizing pooled lymphatic fluid accelerated the course of his infection.

Due to the atypical presentation and the broad nature of known infectious agents, a broad list of differential diagnoses was considered. Those included were multiple arboviruses, rocky mountain spotted fever, toxic shock syndrome (TSS), brown-recluse bite, enteric parasite, viral sepsis, and fever of unknown origin (FUO). This patient was traveling in Virginia when the lesion first appeared; however, the accelerated timeline of symptoms, obvious eschar, and lack of advancing border made rocky mountain spotted fever unlikely [21]. Since the initial wound culture was positive for MSSA, sepsis criteria were met on day 10, and subsequent negative cultures were collected after the initial seven days of TMP/SMX. Staphylococcal TSS was considered [22]. Without the development of worsening abscess/necrosis or multi-organ failure, TSS also was deemed unlikely [23]. Lymphangitis with a necrotic center is consistent with a spider bite, but significant edema and well-healed appearance by day 9 suggested other causes [24]. Travel history merited concern for parasites and arboviruses but was ruled out due to a lack of eosinophilia. While no direct causative agent was found, the resolution of symptoms by day 13 failed to meet the criteria for FUO [25].

At the patient’s three-week follow-up, labs returned significant for CVB antibodies. The final determination was viral sepsis complicated by co-infection with MSSA. After an exhaustive literature review, very few case reports could be found that describe a *Staphylococcus aureus* (MRSA and MSSA) co-infection with CVB. Published reports describe inconsistent and widely variable clinical sequelae that include myocarditis, eczema herpeticum, and sepsis [16,17].

## Conclusions

While both pathogens are common, this case represents how co-infection can lead to various potentially life-threatening presentations. With minimal literature on the subject, we hope to spread awareness and encourage others to present similar cases regarding co-infections of MSSA and CVB.
